# Chelation in Metal Intoxication

**DOI:** 10.3390/ijerph7072745

**Published:** 2010-06-28

**Authors:** Swaran J.S. Flora, Vidhu Pachauri

**Affiliations:** Division of Pharmacology and Toxicology, Defence Research and Development Establishment, Jhansi Road, Gwalior474 002, India; E-Mail: vidhudrde@gmail.com

**Keywords:** chelating agents, combination therapy, oxidative stress, antioxidant, succimer, monoesters, heavy metals

## Abstract

Chelation therapy is the preferred medical treatment for reducing the toxic effects of metals. Chelating agents are capable of binding to toxic metal ions to form complex structures which are easily excreted from the body removing them from intracellular or extracellular spaces. 2,3-Dimercaprol has long been the mainstay of chelation therapy for lead or arsenic poisoning, however its serious side effects have led researchers to develop less toxic analogues. Hydrophilic chelators like *meso*-2,3-dimercaptosuccinic acid effectively promote renal metal excretion, but their ability to access intracellular metals is weak. Newer strategies to address these drawbacks like combination therapy (use of structurally different chelating agents) or co-administration of antioxidants have been reported recently. In this review we provide an update of the existing chelating agents and the various strategies available for the treatment of heavy metals and metalloid intoxications.

## 1. Introduction

Metals are an integral part of many structural and functional components in the body, and the critical role of metals in physiological and pathological processes has always been of interest to researchers. Unfolding the latter has inspired newer therapeutic strategies based on alteration of the metal concentrations in specific body organs and/or entire body evolving branches like metallotoxicology and metallopharmacology. The use of metals to restore the normal healthy physiology of the body either by direct administration of essential metals, or by chelating out excess or toxic metals, or using them as carriers for targeted drug delivery, or for tagging biomolecules for diagnostics, are all techniques that may be classified under the general heading of metallo-pharmacology. However, in the present review we will restrict our discussion to chelation therapy which is an important concept and tool for modifying metal concentrations in the body. Chelation has its origin in the Greek word *chele* that means claw of a lobster, thus depicting the concept of clinging or holding with a strong grip. The term *chelate* was first applied by Sir Gilbert T. Morgan and H. D. K. Drew in 1920. They suggested the term for the caliper-like groups which function as two associating units and fasten to a central atom so as to produce heterocyclic rings [1].

Metal toxicity may occur due to essential metal overload or exposure to heavy metals from various sources. Most metals are capable of forming covalent bonds with carbon, resulting in metal-organic compounds. Metals and metal compounds interfere with functions of various organ systems like the central nervous system (CNS), the haematopoietic system, liver, kidneys, *etc.* Diagnostic testing for the presence of heavy metals, and subsequently decreasing the body’s burden of these substances, should be an integral part of the overall treatment regimen for individuals with a metal poisoning symptomatology or a known exposure to these substances.

## 2. Chelation: Concept and Chemistry

**Figure 1.**
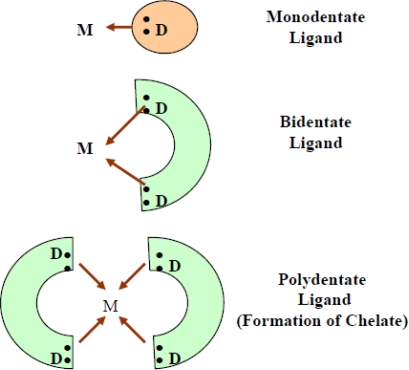
Formation of metal ligand complexes using mono, bi and polydentate ligands.

Although the concept of chelation is based on simple coordination chemistry, evolution of an ideal chelator and chelation therapy that completely removes specific toxic metal from desired site in the body involves an integrated drug design approach. Chelating agents are organic or inorganic compounds capable of binding metal ions to form complex ring-like structure called ‘chelates’. Chelating agents possess “ligand” binding atoms that form either two covalent linkages or one covalent and one co-ordinate or two co-ordinate linkages in the case of bidentate chelates. Mainly atoms like S, N and O function as ligand atoms in the form of chemical groups like –SH, –S-S, –NH_2_, =NH, –OH, –OPO_3_H, or >C=O. Bidenate or multidentate ligands form ring structures that include the metal ion and the two-ligand atoms attached to the metal [2] (Figure 1). Many donors act as bidentate ligands. Five-membered chelate rings are specially stable and they are often formed by ligands with YCCY skeltons such as Y-CH_2_-CH_2_-Y, Y-CO-CH_2_-Y *etc.* where Y is OR, NR_2_, O, S, NR, *etc.* There are also examples of inorganic chelate ligands which form five-membered ring with metal ions. Other types of chelating ligands are possible, like EDTA^4−^, which is a hexadentate ligand. In the simplest case a proton (H^+^) that can absorb the lone pair of electrons of ligand-binding atom(s) of the chelator may be involved in the coordination complex formation. However, the positive charge on proton remains since there is no loss or gain of electrons in the process.

The latter may also be known as the ‘net ionic charge’ of the complex, which plays a crucial role in governing the pharmacokinetic fate and ultimately the toxicological behavior of such complexes *in vivo.* In the biological environment metal cations *viz.* Na^+^, Mg^+^, Cu^+^, Cu^2+^, and Zn^2+^ and specially the transition metals like Mn, Fe and Co may be involved in such complex formation. Although the stability of such complexes varies, the deciding factors are based on the properties of both the chelating agent and the chelated metal. The stability constant of a complex can be quantitatively expressed in equilibrium equation values, which depend on the atomic structure of the chelated metals. For example, the stability constants for different metals with EDTA are on the scale shown in Table 1, where a metal with higher k constant competes for the chelating agent with a metal of lower stability value and ultimately removes the latter.

However, other variables like the number of heterocyclic rings formed and relative concentration also play a role, that’s why Ca^2+^, which is readily available in the body fluids, binds preferentially with Na_2_EDTA in spite of the higher stability constant of Pb. Moreover, in spite of all the known properties desired in an ideal chelator the predictability of the outcome is limited. A chemical entity that qualifies as an ideal chelator *in vitro* might not prove so *in vivo*, either due to the toxicity considerations or due to the presence of endogenous substances (hemoglobin, cytochromes, *etc.*) that can also chelate metal ions and thus offer competition.

Further, pH also is an important factor influencing complex formation and stability. Most chelating agents are unstable at low pH, whereas at high pH metals tend to form insoluble hydroxides which are less accessible to chelating agents. This feature becomes significant in pathological conditions leading to acidosis or alkalosis.

Optimally effective chelation can be achieved by virtue of some combination of the basic properties of both the metal ions, chelating agents and the resulting metal complex. A chelating agent that will occupy more of the coordination positions of a metal ion will generally (but not always) give a complex of greater stability than otherwise. Similarly, whereas the net ionic charge of the chelator defines its absorption, distribution and ability to reach the metal ion for binding; the net ionic charge of the complex decides its elimination from the specific site and excretion from the body. Thus, it is important that a chelator satisfy criteria that allow it to: (1) transport across physiological barriers into compartments where a toxic metal ion is concentrated, (2) form a stable complex with the metal after removing it from the biological chelator, if required at the site and (3) form a chelation complex whose properties render it non-toxic and facilitate its excretion, not only from the site of deposition, but also from the body [3].

## 3. Common Chelating Agents: Pharmacology and Toxicology

**Figure 2.**
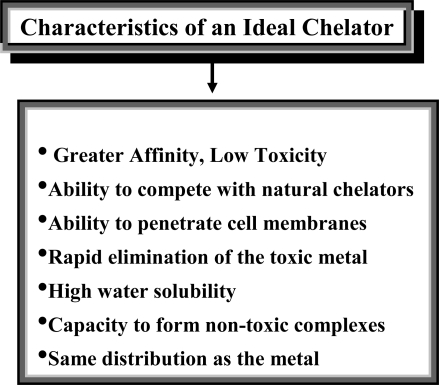
Characteristics of an ideal chelating agent for better chelation of heavy metals.

An ideal chelator should have high solubility in water, resistance to biotransformation, ability to reach the sites of metal storage, retain chelating ability at the pH of body fluids and the property of forming metal complexes that are less toxic than the free metal ion (Figure 2).

**Figure 3.**
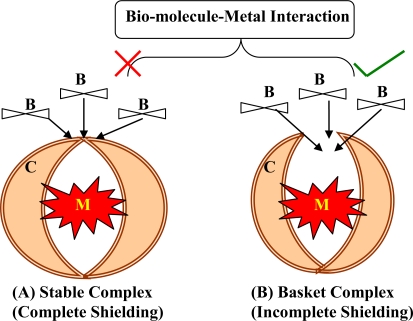
Structures of two different complexes of metals with chelating agents. (A) Stable complex prevent interaction of metal with bio-molecules, (B) Basket complex enhance interaction of metal with bio-molecules. **Symbols used:** B- Bio-molecules; C-Chelating agent; M-Metal.

**Figure 4.**
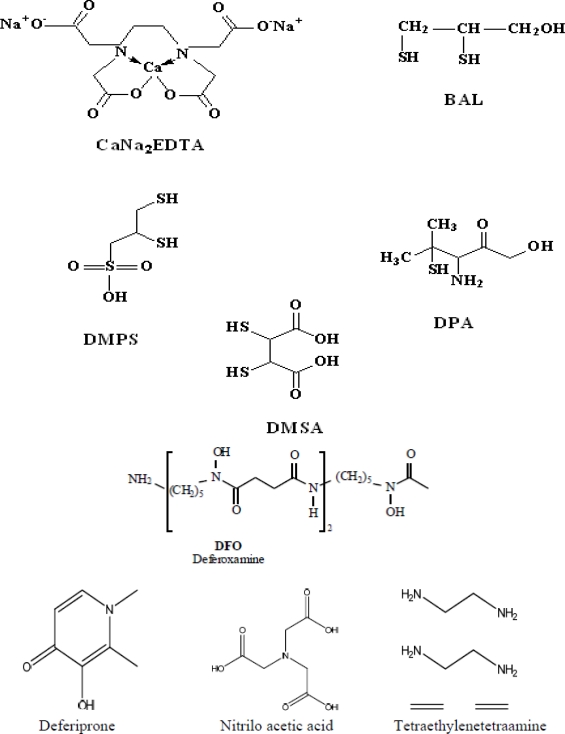
Structures of various chelating agents used to treat cases of heavy metal poisoning.

During Second World War, dimercaprol (also named British Anti-Lewisite or BAL), an organic dithiol compound, was developed as an experimental antidote against the arsenic-based poison gas Lewisite. After World War II, mass lead poisoning was observed in large number of navy personnel, later identified as a result of their jobs repainting the hulls of ships. This introduced the medical use of EDTA as a lead chelating agent. BAL has dominated medical prescriptions for general metal intoxication due to its high efficacy for human arsenic and mercury poisoning. In the 1960s, BAL was modified into meso 2,3-dimercaptosuccinic acid (DMSA), a related dithiol with far fewer side effects. Another dithiol, sodium 2,3-dimercaptopropane 1-sulfonate (DMPS), was introduced as a mercury-chelating agent by researchers in the former Soviet Union. Chelation therapy has historically been used in attempts to reduce the body burden of toxic metals in highly symptomatic patients with elevated biological markers [4–6]. Chelating agents can affect metal toxicity by mobilizing the toxic metal mainly into urine. A chelating agent forming a stable complex with a toxic metal may shield biological targets from the metal ion, thereby reducing the local toxicity [7] (Figure 3). Desferrioxamine (DFOA), an iron chelator, completely covers the surface of Fe^3+^ during complex formation, thereby preventing iron-catalyzed free radical reactions [8]. However, sometimes a chelator may expose the metal to the biological environment and thus increase the toxicity of the metal (Figure 3). Ethylenediamine-tetraacetic acid (EDTA) is not able to shield the surface of the Fe^3+^ ion, but forms an open complex (basket complex), thereby increasing the catalytic capacity of Fe^3+^ for generating oxidative stress [9]. Structures of various chelating agents are presented in Figure 4.

Preferred ligands for soft and borderline ions such as Pb^2+^, Hg^2+^, Cd^2+^ and As^3+^ are thiolates and amines. Consequently, known metal-binding sites in most cases contain Cys or His residues. Many of these sites consist of contiguous short stretches of amino acid sequences that overlap between neighboring peptides with binding activity which are used to tentatively determine major binding motifs. The majority of proteins and peptides that function in the uptake, distribution, storage or detoxification of essential and non-essential metal ions possess one or several metal-binding sites. The -Cys-X-X-Cys- and -Cys-Cys- motifs of various proteins are well known for their heavy metal binding properties [10,11]. It has long been acknowledged that sulfhydryl-containing compounds have the ability to chelate metals. The sulfur-containing amino acids methionine and cysteine, *N*-acetylcysteine, an acetylated analogue of cysteine, the methionine metabolite *S*-adenosylmethionine, α-lipoic acid, and the tripeptide glutathione (GSH) all contribute to the chelation and excretion of metals from the human body.

### 3.1. Calcium Disodium Ethylenediamine Tetraacetic Acid (CaNa_2_EDTA)

Calcium disodium ethylenediamine tetraacetic acid (CaNa_2_EDTA) is the most commonly used chelating agent. It is a derivative of ethylenediamine tetraacetic acid (EDTA); a synthetic polyamino-polycarboxylic acid and since 1950s has been one of the mainstays for the treatment of childhood lead poisoning [12]. The drug has been claimed beneficial in vascular disease since 1955. It is believed that chelation therapy may alter plaque morphology and volume, or improve endothelial function, and suggest that it might replace coronary artery bypass draft surgery [13]. Several theories have been proposed to support latter mainly focusing on calcium chelation. EDTA is said to work in vascular conditions either by removing calcium found in fatty plaques either directly via chelation effect or alternately by stimulating release of hormones that in turn cause calcium removal or lower cholesterol levels. Another theory suggests that EDTA therapy may reduce the oxidative stress injury and inflammation in blood vessel walls [14].

Although a number of trials conducted indicate the utility of CaNa_2_EDTA in coronary heart disease, the evidence in the absence of the controlled trial is not as convincing. In view of reports where symptomatic improvements have been comparable with placebo effects and the risks associated with the therapy, it has been facing criticism [15]. The American Heart Association has stated that “there have been no adequate, controlled, published scientific studies using currently approved scientific methodology to support this therapy for coronary heart disease. Using this form of unproven treatment for coronary heart disease may deprive patients of the well-established benefits from the many other valuable methods of treating these diseases” [13,16] for the program to assess alternative treatment strategies to achieve cardiac health (PATCH) investigators also concluded after a randomized control trial with 84 patient that there is no evidence to support a beneficial effect of calcium chelation therapy with EDTA in patients with ischemic heart disease, stable angina and a positive treadmill test for ischemia. Another sub-study under PATCH rejects the proposed benefits of EDTA in combination with vitamins to improve impaired endothelium-dependent brachial artery flow-mediated vasodilation in patients with coronary artery disease [17]. But recently all randomized clinical trials have been underpowered and the NCCAM and the National Heart, Lung, and Blood Institute launched the Trial to Assess Chelation Therapy (TACT). TACT is the first largest, multicenter study to assess safety and efficacy of EDTA chelation therapy for patients with coronary artery disease. This placebo-controlled, double-blind trial has started recruiting participants, aiming at 2,372 sample size, aged ≥ 50 years with prior myocardial infarction to test whether EDTA chelation therapy, high dose vitamin therapy, or both are effective in secondary prevention. The study started in 2003 and was expected to complete in 5 years [14]. However, trials are ongoing and conclusive results are not expected before June 2012.

CaNa_2_EDTA can be valuable for the treatment of poisoning by metals that have higher affinity for chelating agent than does Ca^2+^. The successful use of CaNa_2_EDTA in the treatment of lead poisoning is due, in part, to the capacity of lead to displace calcium from the chelate. Initially EDTA was introduced as its sodium salt (NaEDTA) which when administered *in vivo* resulted in the urinary excretion of calcium leading to hypocalcaemia with the risk of tetany due to the formation of its calcium complex. To overcome this hazard, CaNa_2_EDTA was introduced for the treatment of lead poisoning. The Pb-EDTA complex has high stability constant thus CaNa_2_EDTA was found to chelate lead from the body fluids, excreting PbNa_2_EDTA leaving Ca behind.

#### 

##### Pharmacological Profile

CaNa_2_EDTA is poorly absorbed in the gastrointestinal tract (<5%) thus can only be administered by parenteral route. Intravenous administration of this drug results in good absorption but is very painful at the injection site. Hence intravenous injection could be given either by diluting in 5% dextrose or saline (iv infusion) [12]. CaNa_2_EDTA is distributed mainly in the extracellular fluids, which limits its capacity to chelate out metals from inside the cells. The latter also contributes to one of its major drawbacks that of redistributing lead from other tissues to the brain. Reporting the same, Flora *et al.* [18] recommended avoiding the use of CaNa_2_EDTA for lead mobilization tests in children. It was suggested that CaNa_2_EDTA when administered in animals chronically exposed to lead, mobilizes the metal deposited in hard tissue for chelation. This then allows mobilized circulating lead to redistribute in soft tissues like the brain and liver in order to achieve equilibrium. CaNa_2_EDTA is not significantly metabolized and is excreted rapidly by glomerular filtration, entirely unchanged in urine, 50% of which appears within one hour [2]. The drug shows an elimination half life of 1.4 to 3 hours in adult and is entirely excreted within 24 hours.

The risks associated with CaNa_2_EDTA therapy are substantial, including renal failures, arrhythmias, tetany, hypocalcaemia, hypotension, bone marrow depression, prolonged bleeding time, convulsions, respiratory arrest, *etc.* [16]. Although the nephrotoxicity by CaNa_2_EDTA is dose dependent and reversible after cessation of therapy yet exceeding maximal daily dose of 75 mg/kg could be fatal. However, there also are reports that highlight the efficacy of EDTA in chronic renal artery diseases [19,20]. Other adverse effects may include fatigue, headache, fever, nasal congestion, lacrimation, mucocutaneous lesions, glycosuria, myalgia, hepatotoxicity, increased urinary frequency, hypotension, abnormal changes in ECG and gastrointestinal symptoms [21]. Prolonged treatment with CaNa_2_EDTA results in depletion of essential metal, especially Zn, Cu and Mn [22]. It has been reported that zinc supplementation during and after chelation is also beneficial [23]. Although zinc depletion by CaNa_2_EDTA therapy is rapidly reversible by zinc supplementation, it is considered to be key mechanism for teratogenic effects of the drug, especially when administered between days 11 to 14 at doses comparable to humans. This allows the use of EDTA as a research tool to study mechanisms and role of zinc dependent molecules at critical periods of pregnancy. CaNa_2_EDTA has the LD_50_ value of 16.4 mmol/kg in mouse [12]. The drug is contraindicated in pregnancy, active renal diseases or anuria, hepatitis, and hypersensitivity to edentate products [21].

### 3.2. Calcium Trisodium DTPA

Calcium or zinc trisodium diethylenetriaminepentaacetate (CaNa_3_DTPA or ZnNa_3_DTPA respectively) have been used against plutonium and other transuranic elements like californium, americium, and curium [24]. The drug is effective against cobalt and zinc poisoning in experimental models [25,26]. Its efficacy against acute cadmium poisoning is promising however is less effective as compare to carbodithioates [27,28].

#### 

##### Pharmacological Profile

Like CaNa_2_EDTA, CaNa_3_DTPA/ZaNa_3_DTPA is poorly absorbed in the gastrointestinal tract, and thus they are parenterally administered and are distributed extracellularly. They may be administered by intravenous or intramuscular routes or by inhalation through a nebulizer [24,29], where the former are painful. CaNa_3_DTPA shows an expected disadvantage of depleting Zn from the system that may be overcome by supplementation or using the zinc salt of the drug. Despite this disadvantage, although CaNa_3_DTPA is still preferred over the Zn form due to its higher efficacy, it is not recommended for prolonged therapy. Although CaNa_3_DTPA is well tolerated, some adverse effects may be observed, including nausea, vomiting, diarrhea, chills, fever and muscle cramps during the first 24 hrs [29]. CaNa_3_DTPA is teratogenic like CaNa_2_EDTA due to its Zn and Mn depletion effect that is confirmed by the teratogenic effects of ZnNa_3_DTPA at a dose 16 times lower than CaNa_3_DTPA. Thus, although the drug is contraindicated during pregnancy ZnNa_3_DTPA may be carefully administered if unavoidable. Other contraindications include prescription to children, in patients with renal insufficiency and bone marrow depression.

### 3.3. D-Penicillamine

d-Penicillamine (DPA; β-β-dimethylcysteine or 3-mercapto-d-valine) is a sulfhydryl containing amino acid and a degradation product of penicillin. Only the d-isomer is used because the l-isomer causes optic neuritis. DPA is used mainly as a chelating agent in heavy metal toxicity *viz.* lead, mercury and copper poisoning (Wilson’s disease) [30].

#### 

##### Pharmacological Profile

DPA is well absorbed via the gastrointestinal tract and can be administered orally or by iv route. It is approximately 50% absorbed orally and mainly follows extracellular distribution. Peak plasma concentration is reached between 1 and 4 hours after oral administration. A minimal drug fraction under-goes hepatic metabolism to disulfides, and most of the drug is excreted unchanged in urine. Elimination half life ranges from 1 to more than 7 hours. Although low adverse reactions have been reported, some serious ones including thrombocytopenia and leukocytopenia (incidence 5–15%), and rarely aplastic anemia may occur. Prolonged DPA treatment may lead to anorexia, nausea and vomiting. The ulcerogenic activity of DPA in rats, which may involve the stimulation of histaminergic (H_1_ and H_2_) receptors, has been reported [31]. Other toxic effects of DPA include gastrointestinal disturbances (10–30%), changes/loss of taste (5–30%), hair loss (1–2%), and partly proteinuria (5–20%) [32]. Severe adverse effects are autoimmune phenomena such as pemphigus, DPA-induced lupus erythematosus, polymyositis/dermatomyositis, membranous glomerulopathy and hypersensitivity pneumonitis [32]. DPA is a well recognized teratogen and lathyrogen that causes skeletal, cutaneous and pulmonary abnormalities [31]. The most important contraindication is in patients allergic to penicillin and in cases of renal insufficiency. Co-administration of DPA to patients receiving gold therapy, antimalarial or cytotoxic drugs, phenyl-butazone, or oxyphenbutazone must be contraindicated since it can result in more serious adverse reactions.

### 3.4. British Anti Lewisite (BAL)

**Figure 5.**
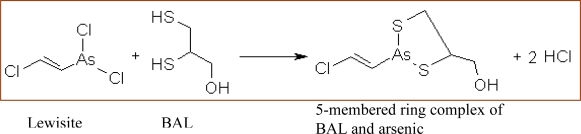
Chemical reaction of lewisite with British Anti Lewisite (BAL) to give a stable 5-membered ring complex.

2,3-Dimercaprol (BAL) is a traditional chelating agent developed by British biochemists at Oxford University during World War II [33]. BAL has a 3-carbon backbone with two sulfhydryl (–SH) groups and a hydroxyl group. It has been used clinically since 1949 in arsenic, cadmium and mercury poisoning. It is an oily, clear, colorless liquid with a pungent, unpleasant typical mercaptan smell. It detoxifies lewisite by forming a five membered stable complex with arsenic (Figure 5). In humans and experimental models, the antidotal efficacy of BAL has been shown to be most effective when administered immediately after the exposure. Besides rapid mobilization of arsenic and mercury from the body, it causes a significant increase in brain deposition of arsenic and mercury compounds [34,35].

#### 

##### Pharmacological Profile

Due to its oily nature, the drug is not absorbed orally and administration of BAL requires deep intra-muscular injection that is extremely painful and allergenic. It was found to mobilize and relocate lead to the brain, increasing its neurotoxic effects [36]. Although treatment with dimercaprol increases the excretion of cadmium, there is a concomitant increase in renal cadmium concentration, so its use should be avoided in cases of cadmium toxicity. The drug has a short half life. Thus, the major drawbacks of BAL include:

1. Low therapeutic index (small margin of safety)
2. Tendency to redistribute arsenic to brain and testes
3. Need for (painful) intramuscular injection
4. Unpleasant odor (rotten eggs)

Other common adverse effects include fever, conjunctivitis (eye inflammation), lacrimation (tearing), constricted feeling (chest, limbs, jaw, abdomen), headache, paresthesias (tingling sensation), tremor, nausea, and pain at the injection site [37]. More serious complications may be infections (abscesses) at the injection site, liver damage, elevated blood pressure and heart rate, and hemolysis (destruction of red blood cells) in patients with glucose-6-phophate deficiency (G6PD) [37].

### 3.5. Meso-2,3-Dimercaptosuccinic Acid (DMSA)

A chemical derivative of dimercaprol which has gained more and more attention, is *meso-*2,3-dimercaptosuccinic acid (DMSA). DMSA is a dithiol compound (containing two sulfhydryl, or –SH, groups) and an analogue of dimercaprol (BAL). It was 40 years ago when initial studies identified DMSA as an effective antidote to heavy metal poisoning. Subsequently DMSA was studied for more than twenty years in the People’s Republic of China, Japan, and Russia before scientists in Europe and the United States “discovered” the substance and its potential usefulness in the mid-1970s [38].

#### 

##### Pharmacological Profile

The hydrophilic nature of DMSA causes considerable absorption in gastro intestinal tract thus possible oral route of administration creates its distinct advantage over BAL. The drug is 95 % plasma protein bound, most likely by virtue of binding on one of its sulfhydryl groups to a cysteine residue on albumin, leaving the other –SH available to chelate metals [39]. DMSA has a large therapeutic window and is the least toxic amongst the dithiol compounds [40]. No significant loss of essential metals like zinc, iron, calcium and magnesium are observed. However, metabolism of copper may be altered in humans after administration of DMSA [41]. A major drawback associated with DMSA is its extracellular distribution, since it is unable to cross the cell membrane. Adverse reaction of DMSA includes gastrointestinal discomfort, skin reaction, mild neutropenia and elevated liver enzymes. The LD_50_ value of sodium salt of DMSA is about 3% greater than that of DMPS [38], in mice it is: 2.4 for i.v., 3.8 for i.m., 4.4 for i.p., and 8.5 gm/kg for oral route.

**Figure 6.**
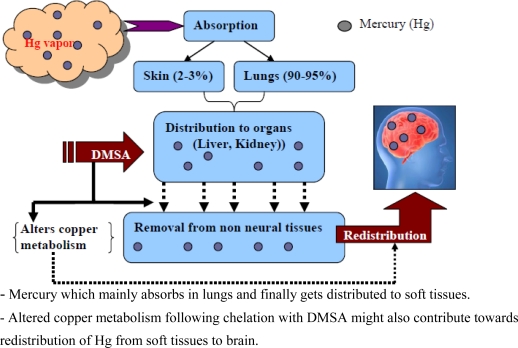
Limitations of DMSA in the treatment of mercury toxicity.

Numerous animal and human studies have shown that DMSA administration increases urinary mercury excretion and reduces blood and tissue mercury concentration [42–44]. Chelation therapy with DMSA must be initiated shortly after exposure to prevent accumulation and avoid toxicity. Studies in animal models have shown that neither DMSA nor any other chelating or mobilizing agents were able to ameliorate the brain burden of mercury [45,46]. The treatment with DMSA after exposure to inorganic mercury caused an increased elevation of mercury into motor axons presumably owing to redistribution of mercury, which was mobilized from non-neural tissues (e.g., kidneys and liver) (Figure 6) [46].

### 3.6. Sodium 2,3 Dimercaptopropane-l-Sulphonate (DMPS)

Sodium 2,3-dimercaptopropane sulfonate (DMPS) is another analogue of BAL. DMPS is not considered as an appropriate drug against lead toxicity. A pilot study of DMPS in lead poisoned children by Gersl and co workers indicates less efficiency than CaNa_2_EDTA and DMSA [47]. DMPS, although known for its antidotal efficacy against mercury, it has also been reported to have limited efficacy for treating lead and arsenic poisoning [18,48]. The drug is registered in Germany for treatment of mercury intoxication, but it is not approved in the United States, so unless special permission is given by the U.S. Food and Drug Administration, it is unlawful for physicians to use it in the United States, nor is it lawful for pharmacies to compound it. Still, DMPS is being illegally used by members of the alternative health industry to treat people allegedly suffering from mercury intoxication, most often claimed to be due to amalgam dental fillings.

#### 

##### Pharmacological Profile

DMPS, being hydrophilic in nature, is mainly distributed in the extracellular space but may enter cells by specific transport mechanisms. Thus, DMPS is known to be distributed both in the extracellular and to small extent in intracellular spaces [2]. DMPS appears to be bio-transformed in humans to acyclic and cyclic disulphides. The drug and its metabolites are rapidly eliminated from the body through the kidneys. It is important to note that this drug does not redistribute arsenic, lead, or inorganic mercury to the brain [49]. No major adverse effects following DMPS administration in humans or animals have been reported [49]. Adverse reactions during treatment with DMPS include gastrointestinal discomfort, skin reactions, mild neutropenia, and elevated liver enzymes. Some patients, especially those with allergic asthma symptoms, may develop hypersensitivity to DMPS [50]. Further, oral administration of DMPS did not adversely affects late gestation, parturition or lactation in mature mice and fetal and neonatal development do not appear to be adversely affected.

### 3.7. New DMSA Analogues

**Figure 7.**
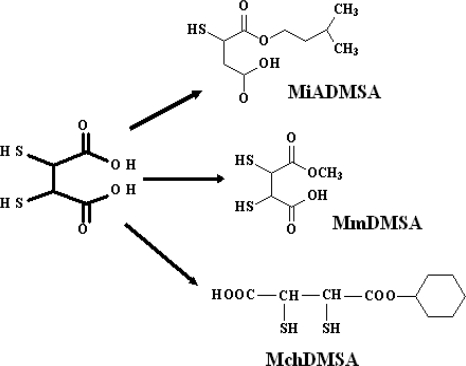
Newly synthesized monoesters of DMSA.

The known chelating agent DMSA has been found to have fewer side effects with greater efficacy. The heavy metal chelating property of DMSA is due to the presence of vicinal dithiol groups that may also act as an oxygen radical scavenger and thus inhibit lipid peroxidation, giving an added advantage of fighting heavy metal induced oxidative stress. However, its hydrophilic properties do not allow it to cross the cell membrane. It was recently observed that esters of DMSA might be more effective antidotes for heavy metal toxicity. A large number of esters of DMSA have been synthesized aiming to for achieve optimal chelation and transport effects, compared to DMSA. These esters are mainly the mono- and diesters of DMSA that have been studied experimentally with the aim of enhancing tissue uptake of chelating agents. In order to make the compounds more lipophilic, the carbon chain length of the parent DMSA was increased by controlled esterification with the corresponding alcohol (methyl, ethyl, propyl, isopropyl, butyl, isobutyl, pentyl, isopentyl and hexyl) (Figure 7). It has also been reported that these mono and diesters have a better potential in mobilizing heavy metal from the tissues in mice [51]. Walker *et al.* [51] studied the effects of seven different monoalkyl esters of DMSA on the mobilization of lead in mice and observed that after a single parenteral dose of the chelator DMSA there was a 52% reduction in the lead concentrations while with the monoesters the reduction varied from 54% to 75%. In most of these published reports, it has been observed that the analogues of DMSA were capable of crossing the biomembranes and were more effective in reducing arsenic burden in acute and sub-chronic intoxication. These studies have also suggested that the monoesters may be preferred over DMSA diesters owing to their higher efficacy against arsenic intoxication and lower toxicity.

#### 3.7.1. Monoisoamyl DMSA (MiADMSA)

Monoisoamyl DMSA was synthesized by controlled esterification of DMSA with isoamyl alcohol. Controlled esterification made MiADMSA, a C_5_ branched chain alkyl monoester of DMSA which was lipophilic in nature as compared to the parent DMSA. Chelation studies with MiADMSA found it to be highly effective in reducing heavy metal burden from various organs in heavy metal exposed animals [52,53].

##### Pharmacological Profile

MiADMSA is a potential drug candidate that is still in its developmental phase thus, its entire pharmacological profile has not been established yet. However, studies to date highlight the efficacy and safety of the new molecule in lead and arsenic acute and chronic toxicity in experimental animals. Results suggest that MiADMSA administration increases the activity of ALAD, a specific lead toxicity biomarker in all the age groups and increased blood GSH levels in young rats. MiADMSA was also found to potentiate the synthesis of MT in liver and kidneys and GSH levels in liver and brain, along with significantly reducing the GSSG levels in tissues. MiADMSA is capable of mobilizing intracellularly bound cadmium [54] and is seen to provide antioxidant functions by removing cadmium from the site of deleterious oxidation reactions. The reduced sulfhydryl groups in this compound may also participate in antioxidant reactions and possibly retard propagation of oxidative damage induced by cadmium. Recently Flora and Mehta [55] suggested reversal of arsenic-induced dysfunctioning with concomitant treatment of MiADMSA *in vitro* and *in vivo*. Employing IR spectroscopy they suggested that MiADMSA binds to arsenic to form an adduct which prevents arsenic from exerting its toxic effect in both models. Mehta and Flora, [56] reported for the first time the comparison of the role in metal redistribution of different chelating agents (three amino and four thiol chelators) on hepatotoxicity and oxidative stress in chelating agent-induced metallothionein in rats. The interaction of MiADMSA and DMSA with essential metals is same. MiADMSA was found to be safe in adult rats followed by young and old rats [57]. Our group reported for the first time the toxicological data of MiADMSA, when administered in male and female rats through the oral as well as the intraperitoneal (i.p.) route (25, 50 and 100 mg/kg for three weeks). The oral route of administration was found better when compared to the i.p. route based on the histopathological studies of the liver and kidney tissues. MiADMSA was seen to be slightly more toxic in terms of copper loss and some biochemical variable in the hepatic tissue in females as compared to male rats [58,59]. The studies concluded that the administration of MiADMSA in female rats is confounded with side effects and may require caution during its use. No observed adverse effect levels (NOAELs) for maternal and developmental toxicity of MiADMSA were 47.5 mg/kg and 95 mg/kg/day, respectively, indicating that MiADMSA would not produce developmental toxicity in mice in the absence of maternal toxicity [60]. Mehta *et al.* [61] have suggested that MiADMSA had no effect on length of gestation, litter-size, sex ratio, viability and lactation. Taubeneck *et al.* [62] showed that the developmental toxicity of DMSA is mediated mainly through disturbed copper metabolism and this may also be true for MiADMSA. It is reported that the toxicity of DMSA with LD_50_ of 16 mmol/kg is much lower than the toxicity of MiADMSA with LD_50_ of 3 mmol/kg, but lesser than BAL (1.1 mmol/kg).

#### 3.7.2. Monomethyl DMSA (MmDMSA) and Monocyclohexyl DMSA (MchDMSA)

MmDMSA has a straight and branched chain methyl group, while MchDMSA has a cyclic carbon chain. Thus, they both have better lipophilic characteristics and might penetrate cells more readily that extracellularly acting chelating agent like DMSA. Both these chelating agents are orally active. Jones *et al.* [54] in their *in vivo* study on male albino mice exposed to cadmium for seven days, observed that administration of MmDMSA and MchDMSA produced significant reductions in whole body cadmium levels. Further, no redistribution of cadmium in brain was observed. The *in vivo* evaluation of these monoesters derived from higher alcohols (C_3_–C_6_ monoesters) proved to have better efficacy as compared to the monoesters derived from lower ones (C_1_–C_2_ monoesters) [54]. Their ability to be administered orally suggests that they may possess considerable advantages in the clinical treatment of lead toxicity however; extensive studies are further required to reach a final conclusion.

### 3.8. Deferoxamine (DFO)

Deferoxamine is a trihydroxamic acid, siderphore secreted by *Streptomyces pilosus*, a fungus. This chelating agent is known for its strong binding affinity for trivalent iron and less affinity for other metals making it a specific chelating agent for iron related diseases such as thalassaemia major as well as aluminium poisoning associated with chronic renal dialysis.

#### 

##### Pharmacological Profile

The absorption of DFO in the gastrointestinal tract is low and thus it is administered mainly through intravenous injection or infusion. It is mainly distributed extracellularly and the protein binding in plasma is low (<10%). It complexes with iron and is excreted rapidly as ferrioxamine, mainly through kidney and one third into bile through faces. Thus, its efficacy also is determined by adequate urine output and may be facilitated by dialysis in case of dysfunctioning. This drug is generally well tolerated with few cases where side effects include opthalamic and auditory toxicity, bacterial and fungal infections, alterations in blood histology, allergic and skin reaction besides few reported adverse effect on lungs, kidney and nervous system [63]. The total intravenous dose of deferoxamine should generally not exceed 80 mg/kg/24 hours. The intramuscular route of administration is not recommended in circulatory shock states.

### 3.9. Deferiprone (L1)

Deferiprone (L1; CP20; 1,2-dimethyl-3-hydroxypyrid-4-one) is an iron chelator and is considered a suitable alternative to deferoxamine in the trasfusional iron overload [64]. It is comparatively less expensive than deferoxamine. Clinical studies suggest that combined administration of deferoxamine and deferiprone for three days per week is very effective in depleting iron overload accompanied by improvement in the cardiac function in transfusion dependent thalassemia patients. It may be noted that deferoxamine is known for reversal of cardiac dysfunction, and deferiprone has also shown cardio-protective effects. These may be attributed to reduction in iron overload however, the specific mechanism is not clear. Besides, due to higher incidences of agranulocyrosis, treatment must be closely monitored [65].

#### 

##### Pharmacological Profile

The major advantages include oral administration and rapid absorption through gastrointestinal tract. L1 is mainly excreted via renal route and has a half life of 47–134 minutes. Dosages of 75–100 mg/kg body weight/day of L1 have been considered effective to maintain stable iron balance and to reduce serum ferritin levels within one year of treatment in iron-overloaded thalassemic patients. L1 is rapidly absorbed mainly from the stomach and reaches the circulation quickly. However, they might be the possibility of food–drug interaction or other gastric factors that delays the appearance of the drug in blood following oral administration. L1 is mainly metabolized as glucoronide conjugates and excreted from urine. Wide variation in the metabolism and clearance of L1 amongst patients have been observed, which mainly depend on iron overload and availability of chelatable iron [66]. The main reported side effects during a deferiprone therapy are arthropathy, gastrointestinal symptoms, headache, and moderate zinc deficiency. These adverse reactions are usually reversed on reducing the dose or discontinuing the drug. Except for severe joint symptoms in few patients, most of the subjects in different clinical trials have been able to continue with L1 therapy for a long term. The most severe, but rare complication following administration of deferiprone is agranulocytosis or neutropenia.

### 3.10. TETA

Tetraethylenetetraamine or trientine is a drug of choice for acute copper intoxication. Although nowadays there is less use of copper utensils, in ancient times the use of copper utensils could lead to extensive exposure to copper. Increased urinary copper excretion has been reported after administration of TETA [2].

#### 

##### Pharmacological Profile

Normally TETA is administered through the oral route but its absorption is relatively poor, as indicated by the as evident from the recovery observed in urine and caracass after administration of an oral dose of C^14^-labeled TETA. Two major metabolites of TETA have been identified, *i.e.*, *N*_1_-acetyltriethylenetetramine (MAT) and *N*_1_,*N*_10_-diacetyltriethylenetetramine (DAT). The former plays a significant role in the molecular mechanism by which TETA extracts copper from the system. The 5–18% of TETA that is systemically absorbed is said to be extensively metabolized, with the majority being excreted in urine as metabolite(s) [67,68]. Wilson’s disease is characterized by disturbance in copper homeostasis which is inherited and leads to progressive increase in accumulation of copper and may even be fatal if not cured. Wilson’s disease was originally treated with DPA but TETA was a better chelator and found to be potentially free of side effects like those of DPA. The recommended dosage to treat Wilson’s disease patients is 0.75–2 g/day. The oral LD_50_ for rats for TETA is 2.5 g/kg body weight and is very close to the recommended dose for treatment of Wilson’s disease.

### 3.11. Nitrilotriacetic Acid (NTA)

Nitrilotriacetic acid (NTA, C_6_H_9_NO_6_), is a polyamino carboxylic acid and is also used as a chelating agent which forms coordination compounds (chelates) with metal ions such as Ca^2+^, Cu^2+^ or Fe^3+^. The uses of NTA are quite similar to EDTA. However, in contrast to EDTA, NTA is easily biodegradable. NTA is available in two forms, one that is a sodium derivative (Na3NTA) and the other iron (FeNTA). Both of these forms have been used as chelating agents [69].

NTA has been used shown to possess the ability to mobilize nickel from brain, heart, kidney and liver of nickel poisoned rats. In a comparative study with six metal binding agents, NTA was highly effective in dialyzing out nickel from the subcellular fractions of liver, kidney and blood corpuscles in rats that were exposed to nickel sulphate [70]. Apart from nickel mobilization, a single dose of NTA has been tried in the removal of manganese from various organs and plasma in rats. Results from these studies have indicated that NTA binds rapidly to Mn and forms stable and diffusible complexes that result in faster excretion of Mn from the rats [71]. Since NTA is considered to be non-mutagenic *in vitro* [72] an epigenetic mechanism is assumed, based on the fact that there is sustained cytotoxicity of zinc ions transfer to the urinary tract [72]. Vacuolated cells of the proximal tubules represent the earliest lesions [73], followed by sloughing, necrosis/apoptosis, and proliferation [74–76]. With chronic application of a high dose level, these alterations lead as a continuum to hyperplastic foci, adenomas, and adenocarcinomas [77]. This ability of both NTA to form tumors cannot be ruled out. Studies however have indicated that tumor formation ability of both NTA is highly route and dose dependented. While FeNTA causes an iron overload and lipid peroxidation in cells and is genotoxic [78,79], Na_3_NTA predominantly binds to zinc and calcium, thereby exerting its toxic effects [72].

## 4. Limitations of Current Chelation Therapy

**Figure 8.**
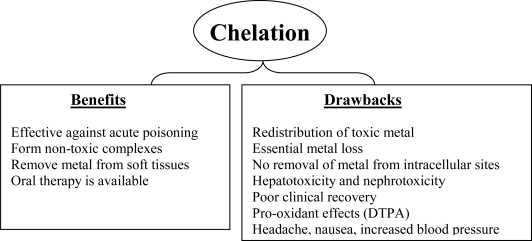
Benefits and drawbacks of chelation therapy.

Most of the currently used chelating agents have serious side effects [80]. Since possible adverse effects and risk associated with conventionally used chelators has already been highlighted in the previous sections, mechanistic limitations are addressed here. CaNa_2_EDTA is a general chelating agent that complexes a wide variety of metal ions and is used clinically despite associated risks. CaNa_2_EDTA cannot pass through cellular membranes and therefore its use is restricted in removing metal ions from their complexes in the extracellular fluid. Similarly conventionally used succimer, DMSA although is considered safer, it shares the limitation of extracellular distribution. The latter renders the drug effective in cases of slow, low dose, chronic metal poisoning (especially lead and arsenic) since metal reaches the cellular compartments behind the physiological barriers including the blood brain barrier. One such classical example was demonstrated during the clinical trial conducted in Bangladesh where DMSA was found ineffective in patients chronically exposed to arsenic [81]. Thus, it is of immediate environmental health concern to identify the limitations of currently available chelating agents and develop new drugs that are more effective in the cases of low, long term exposure to toxic metal. Although treatment with DMSA and DMPS has shown lesser adverse effects, essential metal loss in particular of copper and zinc may be considered as one of the serious notable limitations. Specificity for the target metal may be another domain that needs to be addressed during new drug development. D-Penicillamine (DPA) exhibits disadvantage of possible risk to cause anaphylactic reaction in patient allergic to penicillin. Also prolonged use of DPA may induce several cutaneous lesions, dermatomyosites, adverse effects on collagen, dryness, *etc.* We have summarized the beneficial and the drawbacks associated with chelation therapy are presented in Figure 8.

A retrospective examination of various antidotes reveals that there is no global unanimity of opinion regarding the efficacy of a particular treatment regimen. This is mainly due to different experimental conditions, test protocols and species of animals employed in evaluating various antidotes. Adoption of a particular treatment in a country is dictated by various factors including the regulatory bodies and the legislations. Also, the toxicity of a particular treatment regimen is overlooked in a particular country where as in other countries it is the reason for rejection. The success of any treatment relies on the fact that: (i) it is fast-acting, (ii) has long half and shelf lives, (iii) has minimal side effects and (iv) has ease of application.

The reason to look for newer antidotes are that: (i) there is no effective and safe pre-treatment available which could be instituted as preventive measure against possible arsenic exposure, (ii) the recommended treatments have serious limitations like side-effects or are contraindicated for various instances of heavy metal poisoning, (iii) most of the available treatments are to be given intravenously by a medical practitioner and under no circumstances victim can resort to self aid, (iv) there is no safe and effective oral treatment available and (v) there is no fast acting antidote available which could immediately remove toxic metal from blood and soft tissues. It is thus clear from above that most of the conventional chelators are compromised with many side effects and drawbacks and there is no safe and effective treatment available for heavy metal poisoning.

## 5. Newer Strategies: Combination Therapy

**Figure 9.**
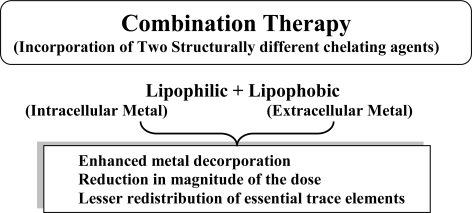
Beneficial effects of combination therapy.

A new trend in chelation therapy is to use two structurally different chelators (Table 2). The concept of combination therapy lies on the fact that two prescribed drug will act through different mechanism of action, thus resulting in additional effect, or sometimes they may support each others’ mode of action leading to synergism. The idea of using combined treatment is based on the assumption that various chelating agents are likely to mobilise toxic metals from different tissue compartments and therefore better results could be expected [18,82]. The combination therapy is an approach to ensure enhanced metal mobilization from the body, reduction in the dose of potential toxic chelators, and no redistribution of toxic metal from one organ to another following chronic metal exposure [18,83–85]. The principle mechanism behind administration of two structurally different chelating agents is illustrated in Figure 9. Flora *et al.* [18] observed that combined administration of DMSA and CaNa_2_EDTA against chronic lead poisoning lead to a more pronounced elimination of lead and better recoveries in altered lead sensitive biochemical variables besides no redistribution of lead to any other organ, was noticed. Co-administration of DMSA and MiADMSA at lower dose (0.15 mmol/kg) was most effective not only in reducing arsenic-induced oxidative stress but also in depleting arsenic from blood and soft tissues compared to other treatments [86].

Treating experimental animals with MiADMSA along with DMSA, we could not only ensure enhanced arsenic elimination but also minimize many serious side-effects, leading to better therapeutic efficacy of the chelators [87]. Since MiADMSA is lipophilic it can chelate intracellular toxic metal and make it accessible to extracellularly available DMSA which may then facilitate rapid excretion of metal from the body. DMSA and MiADMSA in addition to acting as a metal chelator could also act as an antioxidant [88]. The sulfhydryl groups present in these chelators may interact with free oxygen radical or by product of lipid peroxidation like lipid hydroperoxides and aldehydes produced by heavy metal thereby reducing oxidative stress.

**Figure 10.**
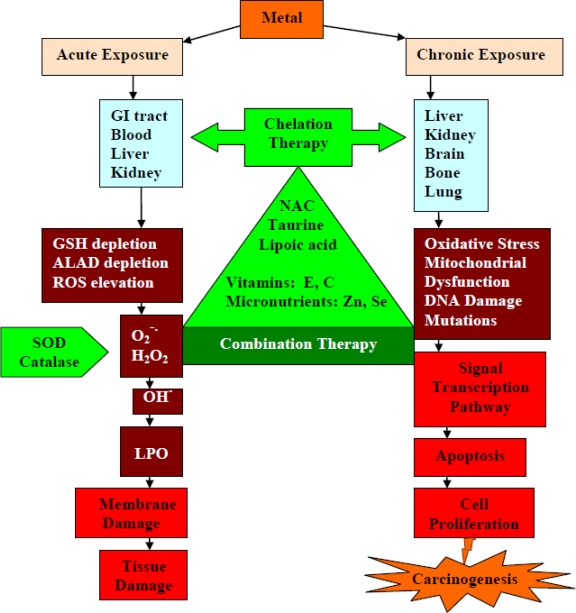
Acute and chronic exposure symptoms of metal toxicity and possible preventive and therapeutic measures against them.

Moreover, the combined administration of DMSA and MiADMSA in chronically arsenic exposed animals was also able to protect against hepatic DNA damage. This observation greatly strengthens the possibility that co-administration of two chelating agents not only gives better efficacy in terms of recovery from arsenic-induced oxidative stress but also helps in reducing the dose of a potentially toxic chelator, thereby minimizing the possible side effects [87]. A reduced dose of chelating agent was found to be beneficial against lead toxicity, with optimum efficacy in the altered biochemical variables and body burden of lead [87]. Recently it has been reported that combined administration of thiol chelator (MiADMSA) and CaNa_2_EDTA is beneficial against chronic lead toxicity in terms of altering neurotransmitters level, neurobehavioral changes, and markers of apoptosis [83]. It was also beneficial in reducing body lead burden and neuronal cell death [83]. Combined administration of DMSA and MiADMSA has found to be highly effective in not only reducing lead burden but also provide better clinical recoveries especially in the brain than monotherapy [89]. Thus, combined administration of DMSA and MiADMSA is recommended for achieving optimum effects of chelation therapy. Figure 10 describes effects of acute and chronic metal exposure and various preventive and therapeutic measures against it.

## 6. Oxidative Stress in Metal Toxicity and the Role of Antioxidants

Arsenic is one of the most extensively studied metals that induce ROS generation and results in oxidative stress. Experimental results show that superoxide radical ion and H_2_O_2_ are produced after exposure to arsenite in various cellular systems [48,86,90]. Shi *et al.* [91] provided evidence that arsenic generates free radicals that leads to cellular damage and death through the activation of oxidative sensitive signaling pathways. ROS play a significant role in altering the signal transduction pathway and transcription factor regulation. Numerous reports have indicated that arsenic affects transcriptional factors either by activation or inactivation of various signal transduction cascades [92,93] Arsenic is known not only to produce ROS but also, nitric oxide (NO•) dimethylarsinic peroxyl radicals (CH_3_)_2_AsOO• and also the dimethylarsinic radical (CH_3_)_2_As• [94,95]. Oxidative DNA lesions induced by arsenic were observed both *in vivo* [96] and *in vitro* [97,98] studies. In a study by Schiller *et al.* [99] it was shown that arsenite can inhibit pyruvate dehydrogenase (PDH) activity through binding to vicinal dithiols in both the pure enzyme and tissue extract. The mechanism of arsenite toxicity was reported owing to its effects on the generation of the hydroxyl radical [100].

Cadmium, unlike other heavy metals is unable to generate free radicals by itself, however, reports have indicated superoxide radical, hydroxyl radical and nitric oxide radicals could be generated indirectly [101]. Watanabe *et al.* [102] showed generation of non-radical hydrogen peroxide which by itself became a significant source of free radicals via the Fenton chemistry. Cadmium could replace iron and copper from a number of cytoplasmic and membrane proteins like ferritin, which in turn would release and increase the concentration of unbound iron or copper ions. These free ions participate in causing oxidative stress via the Fenton reactions [103–105]. Acute intoxication of animals with cadmium has shown increased activity of antioxidant defense enzymes like copper-zinc containing superoxide dismutase, catalase, glutathione peroxidase, glutathione reductase and glutathione-*S*-transferase [106].

Although various mechanisms have been suggested for the toxic effects of Hg, no single mechanism explains all of the pathological outcomes. The chemical reactivity of the metal suggests that oxidative stress might be involved in Hg-induced toxicity. GSH, the primary intracellular antioxidant, was shown to be depleted and to have impaired function in Hg toxicity [107].

Oxidative stress may be considered as one of the prime contributing mechanism in metal toxicity and thus provide a strong rationale for including antioxidants during chelation therapy (Table 2). Antioxidant supplementation with chelating agents has been found beneficial in increasing lead mobilization and providing recovery of altered biochemical variables [108,109]. Combinational therapies with antioxidants like *N*-acetylcysteine (NAC) [53], lipoic acid (LA) [109], melatonin [53], and gossypin [110] have shown considerable promise in improving clinical recoveries in animal models. MiADMSA alone or in combination with captopril was significantly effective in reversing lead-induced apoptosis [111]. We noted significant effect of taurine in depleting blood, liver, kidney and brain lead and arsenic levels, respectively, when co-administered with DMSA or MiADMSA [112,113]. This suggests that the antioxidant capacity of taurine becomes most effective when it is administered along with the thiol chelators or taurine might be facilitating the entry of chelator to the intracellular sites thereby reducing arsenic concentration. NAC is known to have metal-chelating properties and has been used in several clinical conditions [114]. Thiol groups present in NAC act to reduce free radical and provide chelating site for metals. Combined administration of NAC and succimer post arsenic exposure led to a significant turnover in the variables indicative of oxidative stress and removal of toxic metal (arsenic) from the soft organs [115]. Pande *et al.* [116] suggested that NAC could be used both as preventive as well as therapeutic agent along with MiADMSA/DMSA in the prevention or treatment of lead intoxication in rats. Co-administration of vitamin C and MiADMSA in reducing liver and kidney arsenic burden supports the view that vitamin C acts as detoxifying agent by forming a poorly ionized but soluble complex [108]. Recently we have also reported that interaction of non-metal (fluoride) with metalloid (arsenic) also lead to some antagonistic effects [117]. Lipoic acid might also have the capability to interfere with the absorption of arsenic. Beneficial role of LA against lead [109] and GaAs [118] toxicity in terms of lead and arsenic chelation form blood and soft tissues have also been reported. Recently, the clinical importance of herbal drugs has received considerable attention (Table 2). Combination treatment with the thiol chelator and the natural antioxidant C*entella asiatica* proved to be beneficial in the recovery from lead-induced oxidative stress, including the level of biogenic amines and body lead burden as compared with the monotherapy [119].

**Figure 11.**
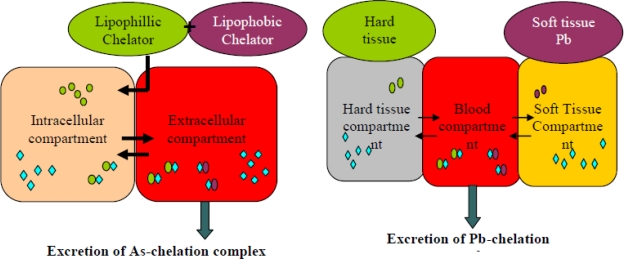
showing possible mechanism involved for the better therapeutic efficacy of combined chelation therapy in lead/arsenic poisoning using two different chelating agents.

Administration of *C. asiatica* during chelation provided more pronounced effects, particularly in the recovery of oxidative stress parameters, suggesting that with the removal of lead from the target tissue, this antioxidant provides effective reversal in the altered parameters indicative of oxidative stress. Number of studies have been reported where a co-administration of a dietary nutrients like a vitamin e.g., thiamine [120], an essential metal *viz.* zinc [121,122] or an amino acid like methionine [123] with a chelating agent lead to many beneficial effects like providing better clinical recoveries as well as mobilization of heavy metal. Supplementation of trace metals has been found to be more effective when given during the course of chelation therapy compared to the chelating agents alone [23,124]. Iron, *in vitro*, is a good chelator of arsenic [125]. Simultaneous supplementation of zinc was found to effectively reverse inhibition of the lead sensitive zinc dependent enzyme ALAD in male Wistar rats [126,127]. When zinc was administered prior to arsenic, there was a reduction in arsenic concentration in several parts of the organism of adult mice, contributing to a decrease in toxicity from the metal [128]. It was also well established that the biosynthesis of metallothionein can be influenced by zinc. The role of zinc supplementation during the course of chelation of lead [23] and cadmium [129,130] has been reported to have many beneficial effects. A more effective removal of hepatic arsenic and recoveries in the arsenic sensitive biochemical indices following combined administration of zinc and MiADMSA [124] may offer an answer to the problem raised with MiADMSA monotherapy [56,57]. Various trends in combination therapy and their beneficial aspects have been summarized in Figure 11.

Phenolic compounds acting as antioxidants may function as terminators of free radical chains and as chelators of redox-active metal ions that are capable of catalyzing lipid peroxidation [131]. Mishra and Flora [132] have also reported that the combined treatment with quercetin and MiADMSA was not only able to chelate arsenic from the cell but also ameliorate oxidant levels, *i.e*., abatement of toxic effects of arsenic. Combined treatments with MiADMSA + NAC and MiADMSA + mannitol were effective both in terms of recovery in parameters of oxidative stress and reduction in blood and tissue cadmium burden [133]. These combined treatments were also effective in partially correcting the cadmium induced loss of liver and brain endogenous zinc. It has been reported that co-treatment with NAC reduces lipid peroxidation and prevents Cd-induced hepatotoxicity [134]. NAC besides preventing hepatotoxicity and reducing the rate of CdMT release from the liver, protect directly by forming a complex with Cd [135], or indirectly by scavenging free radicals [136] or by serving as a cysteine donor for GSH synthesis.

## 7. Therapeutic Recommendations for Heavy Metal Poisoning

Metal poisoning may be acute, sub acute or chronic. Usually acute poisoning is well defined and identifiable, with serious rapid manifestations that may be recovered with immediate medical attention. However, the sub chronic that may convert to chronic metal toxicities may be ill defined as general ill health and not identifiable as any classical syndrome. Moreover, the chronic toxicities may be reversible or irreversible leading to slow development of manifestations like cancer or teratogenic malformations after latent period. The treatment of acute metal poisoning involves emergency medical care that may not be described in the present review. The present review will follow outline of general and specific metal toxicity management. Basic principles of metal toxicity management deals with step by step protocol, for easy understanding that follows as under:

1. Prevention of further metal absorption into the system
2. Elimination of metal from the circulation
3. Inactivation of metal bioavailable in the system

Steps 1 and 2 are more applicable in cases of acute metal poisoning and step 3 is more directed to sub chronic or chronic metal toxicities [137].

### 7.1. Prevention of Further Metal Absorption into the System

The most important and immediate measure is to remove the patient from the exposure. Toxic metals may be absorbed by various routes of exposure including inhalation, dermally or orally. Depending on the intensity and extent of exposure further treatment is decided. In case of high metal exposure as vapours of Hg or concentrated fumes of Pb or As gas, immediate removal of patient not only from the site but removal of clothing, decontamination of skin, eyes, hair and the area around followed by emergency medical assistance may be needed, whereas in case of chronic occupational exposures, or exposure due to lifestyle (household, contaminated drinking water, food, utensils, *etc.*) removal from exposure that may or may not be followed by immediate therapy is needed. The normal excretory system may expel metals to provide a gradual recovery from mild toxicity. In cases of ingestion of toxic metals, acute cases will need stomach emptying within four hours of metal ingestion, or inactivation of metal in the stomach beyond four hours or when gastric emptying is not possible. Various inactivating antidotes, including activated charcoal, milk, egg white, sodium bicarbonate, sodium or magnesium sulphate, Prussian Blue, *etc.* may be used for specific metals that are not discussed herein.

### 7.2. Elimination of Metal from the Circulation

After metal absorption into the circulation in acute cases it may be eliminated from the body to avoid further distribution and penetration in tissues; thus reduce serious damage. Techniques like inducing diureses, modulating urinary pH for metal excretion, employing complexing agents to enhancing faecal excretion for metals undergoing extensive enterohepatic circulation or haemodialysis may be employed.

Although these techniques sounds promising there applicability and efficacy varies depending upon physicochemical properties of metal, route of exposure, intensity and extent of exposure and condition of the patient.

### 7.3. Inactivation of Metal Bioavailability in the System

#### 7.3.1. Lead

The clinical manifestation of lead toxicity is termed ‘plumbism’ and has been known since ancient times. Children are more vulnerable to lead exposure than adults because of the frequency of hand-to-mouth activity (pica), and a higher rate of intestinal absorption and retention. Blood lead has been reported to impair normal metabolic pathways in children at very low levels [138]. Centers for Disease Control and Prevention determined that primary prevention activities in children should begin at blood lead levels (BLLs) > 10 μg/dL [139]. Lead (Pb) binds to sulfhydryl and amide group components of enzymes, altering their configuration and diminishing their activities. It may also compete with essential metallic cations for binding sites, inhibiting enzyme activity, or altering the transport of essential cations such as calcium [83]. Lead produces a range of effects, primarily on the haematopoietic system, the nervous system, and the kidneys. Lead inhibits many stages in the haem synthesis pathway. δ-aminolevulinic acid dehydratase (ALAD), which, catalyses the formation of porphobilinogn from δ-aminolevulinic acid (ALA) and ferrochelatase, which incorporates iron into protoporphyrin [140]. ALA in urine has been used for many years as an indicator of exposure, inhibition of haematopoiesis among industrial workers, and the diagnosis of lead poisoning [140,141]. Ferrochelatase catalyzes the incorporation of iron into the porpohyrin ring. As a result of lead toxicity, the enzyme is inhibited and zinc is substituted for iron, and zinc protoporphyrin concentration is increased [142]. The most vulnerable target of lead poisoning is the nervous system. Lead encephalopathy rarely occurs at blood lead below 100 μg/dL. One of the important mechanisms known for lead induced neurotoxicity is the disruption of calcium metabolism. Oxidative stress, a condition describing the production of oxygen radicals beyond a threshold for proper antioxidant neutralization, has been implicated as a pathologic condition in lead toxicity. Studies have shown that lead causes oxidative stress by inducing the generation of reactive oxygen species (ROS) and weakening the antioxidant defence system of cells [143,144]. Depletion of cells’ major sulfhydryl reserves seems to be an important indirect mechanism for oxidative stress that is induced by redox-inactive metals [145,146].

Other than the supportive therapy for lead exposed subjects, chelation therapy is recommended in symptomatic cases or where blood lead levels are high (50–60 μg/dL) [12]. The recommended dose of CaNa_2_EDTA for asymptomatic adult and pediatric patients with blood lead concentration below 70 μg/dL but above 20 μg/dL is 1,000 mg/m^2^/day given intravenously (*iv*) or by intramuscular (*im*) route. The *iv* administration of CaNa_2_EDTA must always be as infusion over a period of 8–12 hrs, where by *im* route the drug is administered as two doses given at 8–12 hr intervals. However, in patients with blood lead levels higher than 70 μg/dL monotherapy with CaNa_2_EDTA might actually aggravate symptoms. These cases must receive combination treatment of CaNa_2_EDTA with BAL. Similarly, for lead encephalopathy in infants, immediate CaNa_2_EDTA *iv* infusion (1,500 mg/m^2^/day) is administered for five days and complimentary BAL (450 mg/m^2^/day) is recommended four hours before and during CaNa_2_EDTA treatment to avoid lead brain redistribution. Initial therapy increases urinary lead excretion and reduced blood lead burden which is usually followed by a rebound high blood lead concentration at chelation cessation. This happens by virtue of redistribution (mobilization) of metal from reservoirs like skeletal system. Thus, after a two day interval a second course of therapy is prescribed to allow redistribution of lead and replenishment of zinc and other essential metals. Further, anticonvulsive drugs (e.g., Phenytoin) or hyperosmotic (e.g., Dexamethasone) therapy may be added to CaNa_2_EDTA infusion if needed. CaNa_2_EDTA although is effective but is contraindicated in patients with renal insufficiency. Since the lead-EDTA complex is excreted by glomerulus filtration it aggravates chances of renal failure. In adults with lead nephropathy, CaNa_2_EDTA may be prescribed at the dose of 500 mg/m^2^ at various time intervals for five days that may be repeated after a month interval depending upon serum creatinine levels. Dimercaprol was initially prescribed as monotherapy at a dose of 4 mg/kg, *im* at various intervals up to seven days. This is no longer in use due to its adverse effects and availability of safer chelators like DMSA. In less severe cases DMSA (30 mg/kg/day) can be conveniently prescribed for its oral administration and fewer side effects. DMSA has been approved by the U.S. Food and Drug Administration (US FDA) for the treatment of lead intoxication in children with blood lead levels 45 μg/dL. A major advantage of DMSA is that, lead is not redistributed to the brain and other vital organs after its therapy in rats intoxicated with lead [18,84]. Animal studies suggest that DMSA is an effective chelator of lead concentrated in soft tissue but it is unable to chelate lead from bones [142]. Ercal *et al.* [148] indicated that lead induced oxidative stress responded moderately to the treatment with DMSA accompanied by reduction in lead concentration from blood and soft tissue. DMSA for being an antioxidant and a strong lead chelator has been shown to deplete significantly lead from hippocampus leading to recovery in the oxidative stress and apoptosis induced by lead [149]. DMSA binds with lead utilizing –SH groups. Although d-penicillamine also shared advantage of oral administration when prescribed at 250 mg given four times for five days of therapy or 40 mg/kg/day for chronic therapy; it is no longer used for the treatment of lead intoxications as safer profile succimer is available [12]. It is important to note that in cases of occupational lead poisoning, chelation therapy with ongoing exposure is never recommended. Instead patient heavily exposed to lead may be removed from the site and then only then chelation therapy should be administered.

#### 7.3.2. Arsenic

Arsenic is listed as the highest priority contaminant on the ATSDR/EPA priority list of hazardous substances at Superfund sites [150]. Major anthropogenic sources of arsenic in the environment include smelting operations and chromated copper arsenate, a variety of pesticide used in pressure treating wood for construction purposes. Arsenic can be transmitted not just by drinking water, but also by direct exposure to skin and hair. It can also be transmitted through food grains and the possible transmit of arsenic through summer (Boro) rice grown in the Bengal basin is an issue of debate [151]. High levels of arsenic have been found in 10 developing countries, including India [152,153]. In Bangladesh, 57.5% of the studied population had skin lesions caused by arsenic poisoning (*n* = 1,630 adults) [154]. Arsenic toxicity is associated with various hepatic, renal, neurological and skin disorders. At chronic exposure it is known to also produce carcinogenic effects. Arsenic is rapidly and extensively accumulated in the liver, where it inhibits NAD-linked oxidation of pyruvate or α-ketoglutarate. This occurs by complexation of trivalent arsenic with vicinal thiols necessary for the oxidation of this substrate [115]. Dermatological changes following chronic arsenic intoxication are common features and the initial clinical diagnosis is often based on hyper pigmentation, palmar and solar keratosis. Toxic effects of arsenic also include changes in behavior, confusion, and memory loss. Exposure to arsenic in drinking water has been associated with a decline in intellectual function in children. Arsenic is classified as a group 1 carcinogen to humans based on strong epidemiological evidence [155]. Areas in Bangladesh and India with arsenicosis showed high incidences of tumors in local residents [156]. The mechanisms by which arsenic causes human cancers are not well understood. Recent *in vivo* studies indicate that methylated forms of arsenic may serve as co-carcinogens or tumour promoters [157,158]. One of the important mechanisms of arsenic induced disorders is its ability to bind with sulfhydryl group (–SH) containing molecules.

Dimercaprol, at a dose of 3–4 mg/kg *im* every 4 to 12 hrs could be followed by penicillamine for four days, as four divided doses may be given to a maximum of 2 gm/day. Succimer or DMSA has been tried successfully in animal as well as in cases of human arsenic poisoning [159]. DMSA is also efficacious against arsenic toxicity however USFDA has approved DMSA only for lead chelation in children. We also reported significant depletion of arsenic and a significant recovery in the altered biochemical variables of chronically arsenic exposed rats [140]. However in a double blind, randomized controlled trial study conducted on few selected patients from arsenic affected West Bengal (India) regions with oral administration of DMSA suggested that DMSA was not effective in producing any clinical and biochemical benefits or any histopathological improvements of skin lesions [160].

#### 7.3.4. Cadmium

Cadmium (Cd) is one of the most toxic metal ions of our environment and is found in air, food and water. Cd ions are absorbed by most tissues of the body and become concentrated mainly in liver and kidney and it has a long biological half-life of 11 to 20 years in humans [161]. Cadmium is listed by the U.S. Environmental Protection Agency as one of 126 priority pollutants. The most dangerous characteristic of cadmium is that it accumulates throughout a lifetime. Chronic human exposure to Cd results in renal dysfunction, anemia, hepatic dysfunction, osteotoxicities, and cancer in multiple organs, potentially including the kidney [162,163]. Because of its carcinogenic properties, cadmium has been classified as a #1 category human carcinogen by the International Agency for Research on Cancer, Lyon, France [164]. Cadmium is a potent human carcinogen and has been associated with cancers of the lung, prostate, pancreas, and kidney. Cadmium can cause osteoporosis, anemia, non-hypertrophic emphysema, irreversible renal tubular injury, eosinophilia, anosmia and chronic rhinitis. Cd-induced nephrotoxicity is clearly the most important and the most frequently occurring ailment in humans as a result of chronic exposure to the metal [165]. The various toxic effects induced by cadmium and other heavy metals in biological systems might be due to alterations in the antioxidant defense system [166]. Cadmium-induced oxidative damage has been demonstrated by the increased lipid peroxidation and inhibition of enzymes required to prevent such oxidative damage [167]. It has been suggested that the mechanisms of acute Cd toxicity involve the depletion of glutathione and protein-bound sulfhydryl groups, resulting in enhanced production of ROS such as superoxide ion, hydrogen peroxide, and hydroxyl radicals [168,169]. ROS has been implicated in chronic Cd nephrotoxicity [170], immunotoxicity [171], and carcinogenesis [172]. Cd-induced inflammation in the liver is another important mechanism for Cd-induced oxidative stress [173]. Mitochondrion is an important target of Cd toxicity [174]. It has been proposed that Cd initially binds to protein thiols in mitochondrial membrane, affects mitochondrial permeability transition, inhibits respiratory chain reaction, and then generates ROS [175]. Cadmium accumulation in the brain causes behavioral alteration, which is exacerbated in rats fed with low protein diet [176]. Metallothionein (MT), a low-molecular-weight, cysteine-rich, metal-binding protein, has been shown to play a protective role in Cd-induced hepatotoxicity and nephrotoxicity [177].

Effective chelation therapy against cadmium has yet to be identified, but CaNa_2_EDTA has also been recommended with no proven clinical benefits. It is suggested that CaNa_2_EDTA at the dose of 75 mg/kg/day in three to six divided doses for five days (total dose per five days not exceeding more than 500 mg/kg) may be prescribed immediately after Cd exposure. Since decrease in efficacy of cadmium therapy happens in parallel to distribution of metal in the tissue. CaNa_3_DTPA, an effective antidote against cobalt is also found effective against acute cadmium toxicity. However, it is less effective as compared to carbodithioates [27,28]. The usual widely accepted dose of CaNa_3_DTPA or ZnNa_3_DTPA is 1 gm/day in single dose, two to five days a week via *im* administration. Various analogues of carbodithioates including diethyl (De), dimethyl (Dm), and diisopropyl (Di)—dithiocarbamates (DTC) have been investigated for their chelation efficacy against cadmium toxicity. The analogues although were effective but showed greater efficacy with delayed injection indicating interaction with Cd-thionein (bound Cd) rather than free ionic Cd. Further, combination therapy of DeDTC with DTPA caused excretion of increased concentrations of Cd in urine. It was suggested that DTPA acts directly with DeDTC in Cd complexation where DTPA contributes in increased urinary excretion of the complex by virtue of its polarity [167].

#### 7.3.5. Mercury

Mercury is a naturally occurring constituent of the Earth’s crust and is found in several chemical and physical forms. In its elemental (metallic) form, it exists in a liquid state at room temperature and readily volatilizes at standard temperature (0 °C) and pressure (1 atm) to form mercury vapors. In recent years, elemental mercury has proven to be a potential source of toxicosis in children [178,179]. In the environment, humans and animals are exposed to numerous chemical forms of mercury, including elemental mercury vapor (Hg), inorganic mercurous [Hg (I)], mercuric [Hg (II)] and organic mercuric compounds. Elemental mercury can be released from dental amalgam restorations [180] and can then be converted into inorganic mercury in the body which can accumulate in the brain [181]. Metallic mercury vapor is both neurotoxicant and nephrotoxicant. Exposure to significant levels of metallic mercury can result in neurologic, respiratory, renal, reproductive, immunologic, dermatologic, and a variety of other effects [182]. Mercurous and mercuric ions impart their toxicological effects mainly through molecular interactions with sulfhydryl groups on various molecules like GSH, metallothionein (MT) and albumin [183–187]. Mercurials have been reported to cause apoptosis in cultured neurons; however, the signaling pathways resulting in cell death have not been well characterized. It has been reported that skeletal muscle is an important deposit of MeHg [188] and the activated antioxidant defense system of cells provides a compensatory mechanism for HgCl_2_ induced oxidative stress. However, such a phenomenon has not been reported in neurons [189] and hence Hg exhibits a more neurotoxic effect [106].

Dimercaprol and d-penicillamine has been the prescribed chelation agents against inorganic and elemental mercury poisoning. Recommended treatment include, dimercaprol at 5 mg/kg dose *im* for high level symptomatic patients and d-penicillamine at 250 mg/kg orally for asymptomatic patients or incase of low level exposure. Hydrophilic analogues of BAL, DMPS, and DMSA have been successfully tested for treatment of toxicity by mercurial compounds [190]. DMPS has shown effective mobilization of mercury from kidney and reduced its biological half life [191]. DMPS is the drug of choice to reduce the burden of alkylmercury from the body including brain [191]. Further, DMPS is an approved drug in Germany for the treatment of mercury it has also been used for its provocative test. It is important to note that BAL may be contraindicated in organic mercury (phenyl- and alkylmercury) poisoning as the lipid soluble complex formed by it may increase mercury distribution into tissue and brain making it more detrimental.

#### 7.3.6. Iron

Iron is an essential micronutrient utilized in almost every aspect of normal cell function and it is particularly crucial for the conservation of energy. Iron is a well known hepatotoxin. Iron overload is a less frequent condition, but a high content of tissue iron has been associated with several pathological conditions, including liver and heart diseases [193], cancer [194], neurodegenerative disorders [195], diabetes and immunological disorders [196]. Hepatic fibrosis and cirrhosis are the major outcomes of chronic iron overload as well as to repeated blood transfusion [197]. Iron toxicity is thus generally divided into five clinical stages, gastrointestinal toxicity, circulatory shock, relative stability, hepatotoxicity and gastrointestinal scarring. Iron toxicity is associated with primary hemochromatosis, high dietary iron intake and frequent blood transfusion. Oxidative stress is a general condition in hemodialysis patients [198], the periodic intravenous iron injection being a factor contributing to oxidative stress. The gastrointestinal tract is the primary target site, which occur without systemic toxicity. The chief site of systemic toxicity is the heart while liver is susceptible because, unlike other organs it is capable of clearing nontransferrin-bound iron [199]. Toxic shock is the most common cause of death in iron poisoning. At an early stage it is hypovolemic due to significant loss of blood and fluid from gastrointestinal tract. Hepatic necrosis is the next common cause of death. A serum concentration of more than 500 μg/dL is the recommended laboratory indicator. Common symptoms include vomiting within first 30 min to several hours, followed by abdominal pain, diarrhea, hyper-glycemia and fever.

Treatment of iron poisoning involves decontamination of gastrointestinal, supportive care and the administration of a chelating agent. Deferoxamine is the specific and most potent chelator for iron and known for its high affinity for ferric form (Fe^3+^) but has a very low affinity for calcium (Ca^2+^). Deferoxamine needs to be given either through the intramuscular (90 mg to 1 g/kg) or intravenously at a rate of 15 mg/kg/h. Its gastrointestinal absorption is very low. Irrespective of the route of administration the daily dose should not exceed 6 g. Patients should be monitored carefully for gastrointestinal complications and shock after the treatment is over. Oral Deferiprone (1,2-dimethyl-3-hydroxypyrid-4-one) has been shown to be as effective as s.c. desferrioxamine in the removal of iron in human and have a similar but different toxicity profile from desferrioxamine in both animals and humans [200]. There are few recent encouraging developments following the introduction deferiprone in combination with deferoxamine [201]. Variable doses of deferiprone (50–120 mg/kg) and deferoxamine (20–60 mg/kg) have been used to achieve low and safe body iron stores but the effects of these treatments are variables in patients. Various recent studies demonstrated the safety and efficacy of another new iron chelator, Deferasirox in reducing iron burden in iron-overloaded patients. Deferasirox, a tridentate oral chelator approved for the chronic iron overload offers a convenient, effective and promising alternative to deferoxamine [66]. This chelator is likely to be a significant development in the treatment of transfusional iron overload, due to its ability to provide constant chelation coverage and the potential to improve compliance [202].

## 8. Role of Antioxidants during Chelation

Induction of reactive oxygen species by metals and subsequent depletion of antioxidant cell defenses can result in disruption of the pro-oxidant/antioxidant balance in tissues [203]. In the event that oxidative stress can be partially implicated in toxicity of metals, a therapeutic strategy to increase the antioxidant capacity of cells may fortify the long term effective treatment. This may be accomplished by either reducing the possibility of metal interacting with critical biomolecules and inducing oxidative damage, or by bolstering the cells antioxidant defenses through endogenous supplementation of antioxidant molecules. Although many investigators have confirmed metal induced oxidative stress, the usefulness of antioxidants along or in conjunction with chelation therapy has not been extensively investigated yet. Vitamins, essential metals or amino acid supplementation during chelation therapy has been found to be beneficial in increasing metal mobilization and providing recoveries in number of altered biochemical variables [204–206]. These antioxidants (vitamin C and E, α-lipoic acid *etc.*) when given either alone or in combination with a chelating agent proved to be effective in mobilizing metal from soft as well as hard tissue [203]. The important role of heavy metals in oxidative damage suggested a new mechanism for an old problem, whether metals are involved in the oxidative deterioration of biological macromolecules. Although several mechanisms have been proposed to explain the heavy metal induced toxicity, none of the mechanisms have been yet defined explicitly. Indirect *in vivo* evidence of oxidative involvement in metal induced pathotoxicity was demonstrated by alleviation of oxidative stress in the erythrocytes after treatment with thiol containing proven antioxidants, *N*-acetylcysteine and a succimer in arsenic exposed rats [115]. In addition to the role of micronutrients in modifying metal toxicity, these nutritional components can also act as complimentary chelating agents (adjuvants) increasing the efficacy of a known chelator, or by acting independently.

## 9. Conclusions

Metals on the one hand serve as essential components of the normal health physiology yet on the other hand, can cause serious toxic manifestations. Chelation therapy has been the mainstay treatment against metal toxicity. Chelation therapy complexes the metal and allows removal of excess or toxic metal from the system rendering it immediately nontoxic and reducing the late effects. Although a range of metal chelators are now available for toxic metal chelation, development of molecules that may be categorized anywhere close to an ideal chelator is far from reality. Most chelators have the disadvantages of numerous adverse effects, non-specific binding and administration inconvenience. In the world of increasing metal exposure although chelation therapy is an important tool in fighting metal storage disorders yet lack of larger clinical trials still offers controversy on its clinical therapeutic benefits. However, inspite of all the drawbacks it is important to understand the need for more specific and advanced chelation molecules to not only resolve the unanswered poisonings like cadmium toxicity but also to achieve complete clinical recovery in cases of other metal disorders. Further, newer therapeutic strategies should be investigated that may provide better therapeutic outcomes. Employing combination therapy with more than one chelating agent and or prescribing antioxidants or nutraceuticals may be more seriously considered as crucial recommendations of chelation therapy.

## Figures and Tables

**Table 1.** EDTA-metal complex stability constants.

**Metal**	Na	Li	Ba	Sr	Mg	Ca	Mn	Fe	Co	Zn	Cd	Pb	Ni
**K (log)**	1.7	2.8	7.8	8.6	8.7	10.6	13.4	14.4	16.1	16.1	16.4	18.3	18.4

**Table 2.** Therapeutic strategies to address limitations in conventional chelation therapy.

**Therapeutic Strategy**	**Examples**	**Refs.**	**Benefits**

Development of newer chelating, agents	MiADMSA MmDMSA MchDMSA	[86]	- Better therapeutic efficacy. - Access to intracellularly bound metals. - Lesser adverse drug reactions. - Better specificity.
Combination therapy with two chelating agents	DMSA+ MiADMSA MiADMSA+CaNa_2_EDTA	[52,87,89] [85]	- Better chelation efficacy - Removal of intra- and extra-cellular metals. - Prevents metal redistribution - Reduction in dose. - Lesser adverse effects
Chelating agent + Antioxidants	DMSA/MiADMSA + NAC DMSA/MiADMSA + LA MiADMSA+ Quercetin DMSA/MiADMSA + Taurine DMSA/MiADMSA+ Vitamin	[115,207] [109] [132] [112,113] [205,108,120]	- Metal chelation and protection against ROS. - Reestablish Pro/Antioxidant status. - Protects from oxidative stress.
Chelating agent + Micronutrients	DMSA+Zn CaNa_2_EDTA+Zn Fe DMSA+Cu	[16,122,121] [111] [208] [121]	- Modifies toxicokinetics of metals. - Replenish essential metal loss - Cofactors for crucial antioxidant and metabolizing enzymes.
Chelating agent + Herbal extract.	*Centella asiatica* Moringa Oleifera Garlic	[209] [210] [211]	- Plant extracts have been shown to potentiate the efficacy of chelating agents. - Herbal drugs are safer according to traditional claims. - Herbal extracts provide the benefits of natural chelation properties and antioxidant benefits.
